# Supplementary material for: Effectiveness of health education interventions for cervical cancer screening: A quasi-experimental study in Pokhara Metropolitan Slum, Nepal
Source: PLOS Glob Public Health. 2026 Jan 20;6(1):e0005853. doi: 10.1371/journal.pgph.0005853 (PMC12818599; doi:10.1371/journal.pgph.0005853)
Supplement: S1 Protocol — (DOCX) [file pgph.0005853.s002.docx]

**Effectiveness of health education interventions in cervical cancer screening behavior among women in slum areas of Pokhara Metropolitan, Nepal: study protocol for a quasi-experimental study**

**Abstract**

**Introduction**
Cervical cancer is a major public health issue in Nepal, particularly among women in low-resource settings like urban slums. Despite national screening guidelines, uptake remains low due to limited awareness, access barriers, and sociocultural factors. Community-based health education may help improve screening behavior.

**Methods**
A quasi-experimental study will be conducted in two slum areas of Pokhara Metropolitan City. Women aged 30–60 will be randomly selected and assigned to an intervention group (receiving CHW-led education using flipcharts and brochures) or a control group (receiving poster-based information). Data will be collected at baseline, three weeks, and six weeks using structured questionnaires. Outcomes include changes in knowledge, screening uptake, and health beliefs.

**Discussion**
This study will evaluate the effectiveness of CHW-led education compared to standard community information in improving cervical cancer knowledge and screening behavior. Results may guide scalable, community-based interventions to reduce cervical cancer burden in similar settings.

**Trial registration:** The study trial was registered in the Australian New Zealand Clinical Trials Registry (ANZCTR). Trial registration number: ACTRN12625000497404

**Introduction**

Cervical cancer ranks as one of the most prevalent cancers among women worldwide.^[1]^ The majority of cervical cancer-related deaths occur in low- and middle-income countries. ^[1]^ The incidence and mortality rates of cervical cancer vary among the World Health Organization’s (WHO) regions.^[1–3]^ The disparities across regions are attributed to inequalities in access to resources for prevention, screening, and treatment of cervical cancer, as well as social and economic factors such as poverty and gender biases. ^[1,4]^ Cervical cancer predominantly affects younger women, with 20% of children losing their mothers to this disease. ^[5]^

In 2020, cervical cancer was the leading cancer diagnosed among Nepalese women, resulting in the highest number of cancer-related deaths.^[6]^ The age-standardized incidence rate stood at 16.4 cases per 100,000 women, with 2,244 new diagnoses and 1,493 fatalities. ^[6]^ According to the national cervical cancer screening and prevention guidelines, women aged 30-60 are advised to undergo visual inspection with acetic acid (VIA) screening every five years, which initially targeted screening at least 50% of women aged 30 to 60 years, which was later revised to 70% in 2020. ^[7]^ However, by 2019, only 8.2% of women aged between 30 and 49 had undergone cervical screening in Nepal.^[8]^ Several obstacles, including limited awareness, limited trained healthcare professionals, and feelings of embarrassment, hinder the utilization of cervical cancer screening.^[9,10]^ On the other hand, factors like the gender of healthcare providers, effective counseling, and the privacy of screening services are frequently cited as facilitators for screening. ^[8]^

Community Information Channel-led interventions using posters are provided by the health facilities to aware women of cervical cancer. ^[7]^ According to the 2^nd^ amendment of the National Female Community Health Volunteer (FCHV) Program Strategy in 2076 BS, FCHV must have at least passed the SLC. ^[11]^ The fragmented nature of health programs poses a challenge for Female Community Health Volunteers to coordinate activities and deliver the results.^[12]^ So, an assessment of an integrated community health worker (CHW) program model is needed to support the implementation of community-based health interventions for the prevention and control of cervical cancer effectively.

**Methods**

**Study design and setting**

The study will be conducted in two slum areas of Pokhara Metropolitan, Nepal, implementing a quasi-experimental (pre-test-post-test control group) study design involving two groups: intervention (community health worker-led using flipchart and brochure) and control (community information channel led using poster).

**Study participants and sampling procedure**

Women aged 30-60 years from two slum areas of Pokhara Metropolitan City will be recruited as study participants. In the first stage, two slum areas of the Pokhara Metropolitan City will be selected purposively to ensure maximum matching between both slum areas, to ensure participants from both the control and intervention groups share similar characteristics. To control the potential confounding effect of accessibility on screening uptake, slum areas with similar distances to health facilities with VIA services will be selected. In the second stage, one slum area will be conveniently selected as the intervention group, and the other one will be conveniently designated as the control group. In the third stage, the list of women aged 30-60 years in both the selected slum areas will be prepared with the help of the head of the slum areas, and the participants will be randomly enrolled in the study.

**Study process (Flow diagram)**

>33 Slum areas in Pokhara Metropolitan

2 slum areas will be purposively selected

One slum area will be conveniently chosen for control group

One slum area will be conveniently chosen for intervention group

Eligible women of 30-60 years will be completely enrolled (n=155)

Eligible women of 30-60 years will be completely enrolled (n=155)

Baseline data collection

CIC-led Intervention

CHW-led Intervention

1st Follow up, data collection (3^rd^ week)

2^nd^ Follow up, data collection (6^th^ week)

**Figure 1: Study process (flow diagram)**

**Sample size calculation**

The sample size was calculated using screening rate at baseline study=73% (p1), the expected change in proportion up to 87% (p2), ^[13]^ with other values conventionally, as Z_a/2_=1.96 (for 95% CL), Z_β_=0.84 (β=80%), and using the formula for sample size (n)=[(Z_a/2_+ Z_β_)^2^. (p1(1-p1)+p2 (1-p2))]/(p1-p2)^2, we obtained 124.08 for each group. With an attrition rate of 20%, the final sample size for each group will be 155.

**Data collection**

Data will be collected by trained female data enumerators from a public health background through face-to-face interviews using a structured questionnaire adapted from relevant literature and previous studies on similar topics. The questionnaire covers the basic information of the study subjects, such as age, marital status, education level, and occupation. A total of 14 questions (36 scores) will be asked to assess knowledge about cervical cancer disease itself and its risk factors, and prevention and control measures. Behavior related to cervical cancer screening will be assessed by the utilization of a cervical cancer screening question. Pre-testing of the study tool will be done among 31 women aged 30 to 60 years in a slum area within Ward 29 of Pokhara Metropolitan to ensure its reliability and validity.

A baseline study will be conducted in the selected slum areas to assess the knowledge of cervical cancer and demographic and behavioral characteristics of the participants. Post-test will be done among both the study groups (intervention and control) after the third week and the sixth week.

**Inclusion and exclusion criteria**

The inclusion criteria for the study participants will be women aged 30-60 years residing in the slum area, those who have not been diagnosed with cervical cancer, and those who are not planning to migrate to another place in the next 3 months. The exclusion criteria will be women who are currently pregnant and those who have undergone a hysterectomy.

**Intervention**

Ten participants who had at least passed the SLC will be enrolled as community health workers (CHWs) in the intervention group. Training on cervical cancer will be provided to the CHWs by the principal investigator and a nurse using presentation slides. The training will be for one day (4 hours). CHWs will be mobilized in the intervention group using flip charts and brochures. After a day of receiving the training, CHWs will reach the doors of the participants to provide health education on cervical cancer, which will be monitored once by the principal investigator. CHWS will visit the participants just once for an education session, which will take 15 minutes for each participant. CHWs will distribute brochures to every participant in the intervention group. All of the participants of the intervention group will receive the intervention. In the control group, posters will be displayed in two different locations after the baseline study.

**Outcomes**

The primary outcome will be a change in knowledge of cervical cancer. Secondary outcomes include changes in cervical cancer screening uptake, perceived susceptibility, perceived severity, and behavioral intention related to cervical cancer from baseline to midterm and endline assessments.

**Benefits to participants and handling of possible risks**

The study will be done in such a way that no harmful effects are created for the participants. The participants will benefit from the health educational intervention, which focuses on cervical cancer screening behavior and knowledge related to the prevention and control of cervical cancer.

**Ethical considerations**

The ethical approval will be taken from the Institutional Review Committee (IRC), Pokhara University. Written consent from the participants will be obtained before the study begins. Data collection will be conducted in the Nepali language to ensure participants’ understanding. Withdrawal will be accepted at any time during data collection. Anonymity will be maintained by keeping code numbers in questionnaires. Confidentiality will be maintained by using the obtained information for the study only.

**Data Analysis**

Statistical Package for Social Sciences (SPSS v22) will be used to store and analyze quantitative data. A descriptive analysis will be performed, where medians and interquartile range (IQR) will be used to characterize continuous variables, while frequencies and percentages will be used to summarize categorical data. P-values will be calculated, and less than 0.05 will be deemed statistically significant in all two-sided analyses, where needed, using the Pearson's chi-squared test, Mann-Whitney test, Wilcoxon test, and Mc. Nemar test. Effect size (r) in the Mann-Whitney and Wilcoxon tests will be calculated using the formula r=|Z|/√n ^[14]^. The intervals for small effect, intermediate effect, and strong effect are 0.1 to 0.3, 0.3 to 0.5, and 0.5 and higher, respectively ^[15]^. No interim analysis will be performed because of the short time duration.

**Discussion**

The study aimed to assess the effectiveness of community health workers and community intervention channel interventions in cervical cancer knowledge and screening behavior among women of slum areas in Pokhara Metropolitan, Nepal. We expect that the health education interventions will enhance the knowledge on risk factors, symptoms, prevention and control measures, and screening tests of cervical cancer, along with the increased participation in cervical cancer screening.

Previous studies from Nepal and other countries have found that health education interventions are effective in increasing knowledge and screening of cervical cancer. ^[16–18]^ A study from Nepal reported that female community health volunteers (FCHVs) can play a vital role in achieving the national target of cervical cancer screening.^[16]^ In our study, we will train the women within the slum areas who have a minimum of secondary level education. Although the decision to mobilize participants with a minimum of a secondary level of education was made independently by the researcher and the supervisor, it aligns with the criteria outlined in the revised National Female Community Health Volunteer (FCHV) program strategy of 2019^[11]^. According to the strategy, FCHVs are local community women who serve as a bridge between the community and government health facilities^[11]^. So, we will train 10 participants who have the potential and qualifications to become FCHVs in the future.

**List of abbreviations**

CC: Cervical Cancer

CCS: Cervical Cancer Screening

CHW: Community Health Worker

CIC: Community Information Channel

HBM: Health Belief Model

TPB: Theory of Planned Behavior

VIA: Visual Inspection with Acetic Acid

WHO: World Health Organization

**References**

1. World Health Organization. Cervical cancer [Internet]. 2024 [cited 2024 Apr 17];Available from: https://www.who.int/news-room/fact-sheets/detail/cervical-cancer

2. Arbyn M, Castellsagué X, de Sanjosé S, Bruni L, Saraiya M, Bray F, et al. Worldwide burden of cervical cancer in 2008. Ann Oncol [Internet] 2011 [cited 2024 Apr 17];22(12):2675–86. Available from: https://www.sciencedirect.com/science/article/pii/S0923753419343479

3. Shrestha AD, Neupane D, Vedsted P, Kallestrup P. Cervical Cancer Prevalence, Incidence and Mortality in Low and Middle Income Countries: A Systematic Review. Asian Pac J Cancer Prev APJCP [Internet] 2018 [cited 2024 Apr 17];19(2):319–24. Available from: https://www.ncbi.nlm.nih.gov/pmc/articles/PMC5980914/

4. Vu M, Yu J, Awolude OA, Chuang L. Cervical cancer worldwide. Curr Probl Cancer [Internet] 2018 [cited 2024 Apr 17];42(5):457–65. Available from: https://www.sciencedirect.com/science/article/pii/S014702721830134X

5. Guida F, Kidman R, Ferlay J, Schüz J, Soerjomataram I, Kithaka B, et al. Global and regional estimates of orphans attributed to maternal cancer mortality in 2020. Nat Med [Internet] 2022 [cited 2024 Apr 17];28(12):2563–72. Available from: https://www.nature.com/articles/s41591-022-02109-2

6. Lintao RCV, Cando LFT, Perias GAS, Tantengco OAG, Tabios IKB, Velayo CL, et al. Current Status of Human Papillomavirus Infection and Cervical Cancer in the Philippines. Front Med [Internet] 2022 [cited 2024 Apr 17];9:929062. Available from: https://www.ncbi.nlm.nih.gov/pmc/articles/PMC9251542/

7. Family Health Division. National guideline for cervical cancer screening and prevention in Nepal. Kathmandu: Family Health Division; 2010. 2020;

8. Shrestha AD, Andersen JG, Gyawali B, Shrestha A, Shrestha S, Neupane D, et al. Cervical cancer screening utilization, and associated factors, in Nepal: a systematic review and meta-analysis. Public Health 2022;210:16–25.

9. Dangal G, Dhital R, Dwa YP, Poudel S, Pariyar J, Subedi K. Implementation of cervical cancer prevention and screening across five tertiary hospitals in Nepal and its policy implications: A mixed-methods study. PLOS Glob Public Health [Internet] 2024 [cited 2024 Apr 17];4(1):e0002832. Available from: https://journals.plos.org/globalpublichealth/article?id=10.1371/journal.pgph.0002832

10. Baral G, Baral R. Cervical Cancer Screening and HPV Vaccination in Nepal. South Asian J Cancer [Internet] 2023 [cited 2024 Apr 17];12(1):53–4. Available from: https://www.ncbi.nlm.nih.gov/pmc/articles/PMC9966170/

11. DoHS. National Female Community Health Volunteer Program Strategy. 2019.

12. Khatri RB, Mishra SR, Khanal V. Female Community Health Volunteers in Community-Based Health Programs of Nepal: Future Perspective. Front Public Health [Internet] 2017 [cited 2024 Dec 22];5. Available from: https://www.frontiersin.org/journals/public-health/articles/10.3389/fpubh.2017.00181/full

13. Paskett ED, Tatum CM, D’Agostino R Jr, Rushing J, Velez R, Michielutte R, et al. Community-based Interventions to Improve Breast and Cervical Cancer Screening: Results of the Forsyth County Cancer Screening (FoCaS) Project1. Cancer Epidemiol Biomarkers Prev 1999;8(5):453–9.

14. Rosenthal R, DiMatteo MR. Meta-Analysis: Recent Developments in Quantitative Methods for Literature Reviews. Annu Rev Psychol [Internet] 2001 [cited 2025 Jun 1];52(Volume 52, 2001):59–82. Available from: https://www.annualreviews.org/content/journals/10.1146/annurev.psych.52.1.59

15. Cohen C. Statistical Power Analysis for the Behavioral Sciences, utg. 2. N Y 1988;

16. Dhoj Shrestha A, Gyawali B, Shrestha A, Shrestha S, Neupane D, Ghimire S, et al. Effect of a female community health volunteer-delivered intervention to increase cervical cancer screening uptake in Nepal: a cluster randomized controlled trial. Prev Med Rep [Internet] 2022 [cited 2025 Jul 2];29:101948. Available from: https://www.sciencedirect.com/science/article/pii/S2211335522002558

17. Abiodun OA, Olu-Abiodun OO, Sotunsa JO, Oluwole FA. Impact of health education intervention on knowledge and perception of cervical cancer and cervical screening uptake among adult women in rural communities in Nigeria. BMC Public Health [Internet] 2014 [cited 2025 May 21];14(1):814. Available from: https://doi.org/10.1186/1471-2458-14-814

18. Rosser JI, Njoroge B, Huchko MJ. Changing knowledge, attitudes, and behaviors regarding cervical cancer screening: The effects of an educational intervention in rural Kenya. Patient Educ Couns [Internet] 2015 [cited 2025 May 21];98(7):884–9. Available from: https://www.sciencedirect.com/science/article/pii/S0738399115001329
