# Supplementary material for: Effectiveness of health education interventions for cervical cancer screening: A quasi-experimental study in Pokhara Metropolitan Slum, Nepal
Source: PLOS Glob Public Health. 2026 Jan 20;6(1):e0005853. doi: 10.1371/journal.pgph.0005853 (PMC12818599; doi:10.1371/journal.pgph.0005853)
Supplement: Attachment — Submitted filename: Response to reviewers.docx [file pgph.0005853.s005.docx]

**Response to reviewers**

Dear Editor,

We sincerely thank you and the reviewers for the constructive feedback provided on our manuscript. We greatly appreciate the time and effort invested in reviewing our work and agree that the comments will significantly strengthen the quality of our paper. Below, we provide a detailed, point-by-point response to the reviewers’ comments and describe the revisions we have made in the manuscript.

**Reviewer #1: Comment 1**: This appears to be a well-designed (but non-randomised) study the results of which would be of considerable interest. Unfortunately, the statistical approach to analysis is far from optimal and mostly inappropriate. Although not immediately statistical, the major shortcoming is that the endpoints used for the evaluations are not defined. Thus, although the authors state (lines 32-33), “The intervention group demonstrated significant improvements in knowledge, perceived susceptibility, perceived severity, behavioral intention, and screening uptake ...” no scale for each outcome measure is defined. Without this information, further review is not useful.

**Response**: Thank you for highlighting this. In the revised Methods section, we now provide a clear definition of each outcome variable (knowledge, perceived susceptibility, perceived severity, behavioral intention, and screening uptake). Specifically,

- **Knowledge:** measured using 14 structured questions (maximum score: 36) covering cervical cancer introduction, risk factors, symptoms, screening methods, and prevention/control measures. Higher scores indicated greater knowledge.
- **Screening uptake:** assessed by self-report of having undergone cervical cancer screening in the past 5 years (yes/no).
- **Perceived susceptibility:** measured with a single-item Likert scale question (1 = very unlikely, 5 = very likely).
- **Perceived severity:** measured with a single-item Likert scale question (1 = no impact, 5 = extreme impact).
- **Behavioral intention:** assessed on a probability scale from 0% (not at all likely) to 100% (certain).

“We have now clarified these points in the Outcome Measures subsection of the Materials and Methods section (Lines 203–216, revised manuscript).”

**Reviewer #1: Comment 2**: My advice to the authors is to find help from an experienced medical statistician in preparing a revision of this paper

**Response**: We sincerely appreciate this constructive suggestion. The statistical aspects of the study were rigorously assessed by a **biostatistician and public health faculty** during both the internal and external defense of the corresponding thesis at the University, and we have duly acknowledged him. For the revision of this manuscript, the analyses were reassessed and finalized by co-authors with expertise in public health and biostatistics**: Mr. Chiranjivi Adhikari** (Assistant Professor, H-index; Scopus: 7 (Scopus ID: 57211843997), Google Scholar:12) and Dr. Hari Prasad Kaphle (Associate Professor, H-index; Scopus: 6 (Scopus ID: 56366264500), Google Scholar: 16). Their input ensured that the statistical methods are appropriate, rigorous, and clearly presented. These updates are detailed in the Statistical Analysis subsection of the Materials and Methods section (Lines 222–236, revised manuscript).

**Reviewer #2**: **Comment 1**: My major concern is the design of the study, where you compare two different interventions in two separate populations. From the start you show with your analysis that the populations differ significantly on core aspects and consequently it is hard to conclude whether differences in outcomes are only based on interventions or may at least partly be attributed to differences in populations. As design cannot be changed, I recommend that you discuss this weakness in depth.

**Response:** We thank the reviewer for highlighting this important limitation. We have now addressed it in the Discussion section of the revised manuscript: *"A key limitation of this study is the comparison of two different interventions in separate populations that differed in ethnicity, major source of health information, and income. Consequently, differences in outcomes may not be attributed solely to the interventions, as part of the observed effects may reflect inherent differences between the populations."* (Lines 445–449, revised manuscript)

**Reviewer #2**: **Comment 2**: You test two different interventions which are very different in use of resources etc. It is mandatory that the interventions are described in more details. For example - the written material could be inappropriate and explain the lack of effect. Have You performed any testing ahead of using the intervention?

**Response:** We thank the reviewer for this valuable suggestion. The training materials and intervention packages were pilot-tested in a small group in the slum areas of Ward 29 to evaluate feasibility and acceptability. Specifically:

- Five women were trained using the presentation slides, and all scored above 90% on the post-training assessment, demonstrating adequate knowledge acquisition.
- These trained CHWs were then mobilized in their local areas to provide health education to two eligible participants each, using flip charts and brochures. Significant improvements in participants’ knowledge were observed after receiving the education sessions, indicating the effectiveness of the CHW-led intervention.
- The effectiveness of the posters was not tested during the pilot, as they were adopted directly from the National Health Education, Information and Communication Centre (NHEICC), Nepal, and are already widely used in health facilities.
- Feedback from both CHWs and participants was used to refine the intervention package, which was then finalized for implementation.

These details are now included in the *Development and testing of intervention packages* subsection of the Methods (Lines 159–170, revised manuscript).

**Reviewer #2**: **Comment 3**: Moreover, the questionnaire must be presented with more details to provide the reader with sufficient information for evaluating the results.

**Response:** We thank the reviewer for this suggestion. The Methods section has been updated to provide full details of the outcome measures, including the questionnaire items and scoring:

- **Primary outcome:** Change in knowledge of cervical cancer.
- **Secondary outcomes:** Changes in cervical cancer screening uptake, perceived susceptibility, perceived severity, and behavioral intention from baseline to midterm and endline assessments.
- **Knowledge:** Measured using 14 structured questions (maximum score: 36) covering cervical cancer introduction, risk factors, symptoms, screening methods, and prevention and control measures. Higher scores indicated greater knowledge.
- **Screening uptake:** Measured by asking whether participants had undergone cervical cancer screening within the past five years; those who had were classified as having screening uptake.
- **Perceived susceptibility:** Assessed with a single-item question: *“How likely do you think it is that you could develop cervical cancer?”* Responses were rated on a 5-point Likert scale (1 = very unlikely, 5 = very likely), with higher scores indicating greater perceived susceptibility.
- **Perceived severity:** Measured with the question: *“If you were to get cervical cancer, how much of an impact would it have on your life?”* Responses were rated on a 5-point Likert scale (1 = no impact, 5 = extreme impact), with higher scores reflecting greater perceived severity.
- **Behavioral intention:** Assessed by asking participants to estimate the likelihood of undergoing cervical cancer screening in the future, rated on a probability scale from 0% (not at all likely) to 100% (certain).

These details are now included in the *Outcome Measures* subsection of the *Materials and Methods* section (Lines 199–216, revised manuscript).

**Reviewer #2**: **Comment 4**: Given that the two interventions are very different with regards to use of resources I recommend that You also describe the intervention provided by Health Care Workers in more detail and compare the two interventions in this perspective.

**Response:** We thank the reviewer for this important suggestion. The Methods section has been updated to provide a detailed description of both interventions and their relative use of resources:

- **CHW-led intervention:** After one day of training, community health workers (CHWs) visited the homes of every participant in the intervention group to provide a single health education session on cervical cancer. Using standardized flip charts, CHWs delivered information on risk factors, early warning signs, prevention and control measures, the importance of screening, available screening methods, and the benefits of early detection and treatment. Each session lasted approximately 15 minutes and was designed to be interactive, allowing participants to ask questions. CHWs also distributed brochures summarizing key messages. The intervention was monitored by the principal investigator to ensure fidelity. All participants in the intervention group received this session before the midterm survey.
- **CIC-led intervention:** In the control group, a minimal CIC-led intervention was provided to uphold participants’ rights. After the baseline survey, informational posters focusing on cervical cancer prevention and screening were displayed at two locations within the study site. The posters included pictorial and descriptive information in Nepali and remained in place throughout the study period.
- **Comparison of resource use:** The CHW-led intervention required substantially greater human and material resources compared to the CIC-led intervention, which consisted only of poster display.

These details are now included in the *Interventions* subsection of the *Materials and Methods* section (Lines 177–197, revised manuscript).

**Reviewer #2**: **Comment 5**: In general, I suggest a condensation of the manuscript. Many results are given in both Tables and repeated in the result section - please choose and I suggest to reduce the number of Tables.

**Response:** We thank the reviewer for this helpful suggestion. In the revised manuscript, we have reduced the number of tables from seven to four: Table 2 and Table 6 were merged into Table 2, Table 3 and Table 7 were merged into Table 3, and Table 4 and Table 5 were merged into Table 4. Additionally, the descriptions in the Results section have been condensed to remove repetition and improve clarity while retaining all key findings.

**Reviewer #2**: **Comment 6**: Several times you refer to the WHO 90-70-90 strategy which is your outcome goal. However, the WHO strategy aims to have 70% of younger women screened by HPV whereas you screen by VIA which is no longer recommended by the WHO. I am aware that VIA is the only possibility in some countries by I think you should emphasize the deviation from the official recommendations.

**Response:** We thank the reviewer for this important observation. We have clarified in the Discussion section that this study used visual inspection with acetic acid (VIA) as the screening method, whereas the WHO 90-70-90 strategy recommends HPV-based screening. VIA was chosen due to feasibility and resource constraints in the study setting; therefore, comparisons with the WHO target should be interpreted cautiously, as the screening method used deviates from current official recommendations. This clarification has been added at the end of the Discussion section (Lines 455–459, revised manuscript).
