# Supplementary material for: Effectiveness of health education interventions for cervical cancer screening: A quasi-experimental study in Pokhara Metropolitan Slum, Nepal
Source: PLOS Glob Public Health. 2026 Jan 20;6(1):e0005853. doi: 10.1371/journal.pgph.0005853 (PMC12818599; doi:10.1371/journal.pgph.0005853)
Supplement: Attachment — Submitted filename: Response to reviewers 2.0.docx [file pgph.0005853.s006.docx]

**Response to reviewers**

Dear Editor,

Thank you very much for the opportunity to revise our manuscript. We are grateful to you and the reviewers for the thoughtful and constructive comments provided during this second round of review. We appreciate the time and expertise devoted to evaluating our work, and we are pleased to note that the study design and relevance of the findings were acknowledged.

We also appreciate the reviewer’s observation that the previously noted concern regarding undefined endpoints has now been adequately addressed. In this revised version, we have focused on strengthening the statistical analysis in line with the reviewer’s recommendations. Below, we respond to each comment in detail and outline the corresponding revisions made to the manuscript.

**Reviewer #1: Comment 1:** Table 1 and elsewhere: The IQR is not a very useful format for describing ages groups. Better to quote the median, minimum and maximum values in the two intervention groups. This applies to age and monthly income.

**Response:** Thank you for this valuable suggestion. Following your recommendation, we have added the minimum and maximum values for age and monthly income in both intervention groups. The table now reports the median, minimum, and maximum values instead of the IQR. We have also revised the table description to reflect these changes clearly. *(Lines: 269-290)*

**Reviewer #1: Comment 2:** Table 1 and elsewhere: P-values should be quoted to two significant figures, for example ‘0.114’ becomes ‘0.11’ and ‘0.845’ becomes ‘0.85’ while ‘0.070’ stays as it is.

**Response:** Thank you for the helpful comment. We have revised the statistical values throughout the manuscript to follow the two-significant-figure format as recommended. All relevant tables and text have been updated accordingly.

**Reviewer #1: Comment 3:** Table 2: If for example, the endpoint Knowledge cannot be assumed to have a Normal distribution, then median, minimum and maximum of each group should be given, together with the difference between the two medians (this is not a straight forward calculation of median1–median2), and the corresponding 95% confidence interval (CI). A CI is easier to interpret than an effect size (lines 234-237). These calculations require appropriate statistical software, but I don’t know if this is available on SPSS. On the other hand, if the data can be assumed approximately Normal, means can be used, and things become a lot easier.

**Response:** Thank you for this important comment. As suggested, we removed effect sizes for non-normally distributed outcomes and now report medians, minimums, and maximums for each group, along with the median difference and 95% confidence interval. These analyses were conducted using R (Version: RStudio/2025.05.1+513), which enables non-parametric bootstrapping for robust CI estimation. Accordingly, the results section in the abstract *(Lines: 37-39; 41-44)*, methodology *(Lines: 237-240)*, table description, and Table 2 *(Lines: 291-308)* have been revised to reflect these changes. We believe this approach improves the clarity and interpretability of the results.

**Reviewer #1: Comment 4:** Table 3: This overlaps with the information in Table 1. The latter compares between interventions the former within interventions. These two would be better combined in a trend analysis. For example, from Table 1 the mean value of the three Intervention medians for Knowledge is (2 + 7.5 +8)/3 = 5.83 and that for the Control (3 + 5 +5)/3 = 4.33. So, the trend upwards over time is greater in the Intervention and this is really what is required for Table 3. Strictly this type of calculation should be made for each subject and then averaged within each group and then compared between two groups.

**Response:** We thank the reviewer for the insightful comment regarding Table 3. Following your suggestion, we have calculated each subject’s average score across the three time points for knowledge, perceived susceptibility, perceived severity, and behavioral intention. These per-subject averages were then summarized within each group, and overall medians with 95% confidence intervals were computed and compared between the intervention and control groups using the Mann-Whitney U test.

The revised Table 3 now shows that the intervention group had higher overall medians compared with the control group: knowledge (median = 6; 95% CI: 5.00–7.33 vs median = 4.33; 95% CI: 3.67–5.00; p < 0.01), perceived susceptibility (median = 3; 95% CI: 3.00–3.33 vs median = 2.67; 95% CI: 2.33–3.00; p < 0.01), and behavioral intention (median = 50; 95% CI: 50–63.33 vs median = 36.67; 95% CI: 33.33–46.67; p < 0.01). For perceived severity, the medians were the same (median = 4.33), but the confidence intervals differed slightly (intervention 95% CI: 4.33–4.67 vs control 95% CI: 4.00–4.33), and the difference was statistically significant (p = 0.03). *(Lines: 309-321)*

These results clearly illustrate the greater upward trend over time in the intervention group compared with the control group. Accordingly, we believe the revised Table 3 now better represents the longitudinal trend of the outcomes, as suggested.
