# Supplementary material for: Effectiveness of health education interventions for cervical cancer screening: A quasi-experimental study in Pokhara Metropolitan Slum, Nepal
Source: PLOS Glob Public Health. 2026 Jan 20;6(1):e0005853. doi: 10.1371/journal.pgph.0005853 (PMC12818599; doi:10.1371/journal.pgph.0005853)
Supplement: Attachment — Submitted filename: Response to reviewers 3.0.docx [file pgph.0005853.s007.docx]

**Response to reviewers**

Dear Editor,

Thank you very much for the opportunity to revise our manuscript. We are grateful to you and the reviewers for the thoughtful and constructive comments provided during this third round of review. We appreciate the time and expertise devoted to evaluating our work.

Below, we respond to the comment in detail and outline the corresponding revisions made to the manuscript.

**Reviewer #1: Comment 1:** The authors have made important changes to the manuscript. One minor but important problem remains.

My previous review stated: “2. Table 1 and elsewhere: P-values should be quoted to two significant figures, for example, ‘0.114’ becomes ‘0.11’ and ‘0.845’ becomes ‘0.85’ while ‘0.070’ stays as it is.” This does not mean 2 decimal places. So, the many places where p < 0.01 are quoted should be replaced by p= 0.01x, where x is the second significant figure. For example, p < 0.01 could imply that p = 0.009 or even 0.005 which should then be reported as 0.009X and 0.005X

**Response:** Thank you for the clarification regarding the reporting of p-values to two significant figures. We have revised the manuscript accordingly, and the SPSS analysis files were revisited to confirm all p-values. All instances where p-values were previously reported as p < 0.01 have now been replaced with exact p-values reported to two significant figures (e.g., 0.014 in Table 2, 0.017 in Table 4) as recommended. For extremely small p-values (< 0.001), where the statistical software reported values in scientific notation without meaningful decimal precision, results have been conservatively reported as p < 0.001, consistent with scientific papers.

*These revisions have been applied consistently across all tables and the main text.*
